# Evaluation of the Adult Migrant English Program for the Australian Government Department of Home Affairs

**DOI:** 10.23889/ijpds.v9i5.2563

**Published:** 2024-09-18

**Authors:** Francis Mitrou, Ha Trong Nguyen

**Affiliations:** 1 The University of Western Australia; 2 Telethon Kids Institute

## Objectives and Approach

The Adult Migrant English Program (AMEP) is a free service to help eligible migrants and humanitarian entrants with low English proficiency to improve their English skills and elevate their participation in Australian society. In partnership with AMEP funder, the Australian Government Department of Home Affairs, we have completed a longitudinal research study analysing the effectiveness of AMEP by linking participant information for over 400,000 AMEP clients with administrative data from social services, taxation, and the national census, from 2003 – 2019. This is the first time AMEP participant data has been linked with other data assets and represents the most comprehensive evaluation of AMEP since program inception in 1948.

## Results

AMEP participation was associated with improved English proficiency of clients, especially when clients studied for longer periods. Migrants with higher levels of English on-entry had better labour market outcomes, higher income levels (male and female), lower rates of public housing tenancy, and were less likely to receive income support in the years following program exit.

## Conclusions

While the design does not support true causal inference, this evaluation provides good evidence that AMEP is effective at improving English proficiency and suggests AMEP participation can improve a range of economic and social outcomes for eligible migrants with low English proficiency.

## Implications

The launch of these results coincided with new investment in AMEP and a broadening of eligibility criteria to make AMEP available to a wider range of migrants with low English proficiency.

